# Fertilization of Microbial Composts: A Technology for Improving Stress Resilience in Plants

**DOI:** 10.3390/plants12203550

**Published:** 2023-10-12

**Authors:** Temoor Ahmed, Muhammad Noman, Yetong Qi, Muhammad Shahid, Sabir Hussain, Hafiza Ayesha Masood, Lihui Xu, Hayssam M. Ali, Sally Negm, Attalla F. El-Kott, Yanlai Yao, Xingjiang Qi, Bin Li

**Affiliations:** 1 Xianghu Laboratory, Hangzhou 311231, China; temoorahmed@zju.edu.cn (T.A.);; 2 Institute of Biotechnology, Zhejiang University, Hangzhou 310058, China; nomansiddique834@gmail.com; 3 Department of Bioinformatics and Biotechnology, Government College University, Faisalabad 38000, Pakistan; mshahid@gcuf.edu.pk; 4 Department of Environmental Sciences, Government College University, Faisalabad 38040, Pakistan; sabirghani@gmail.com; 5 Department of Plant Breeding and Genetics, University of Agriculture, Faisalabad 38000, Pakistan; 6 MEU Research Unit, Middle East University, Amman 11831, Jordan; 7 Institute of Eco-Environmental Protection, Shanghai Academy of Agricultural Sciences, Shanghai 201403, China; xulihui@saas.sh.cn; 8 Department of Botany and Microbiology, College of Science, King Saud University, Riyadh 11451, Saudi Arabia; hayhassan@ksu.edu.sa; 9 Department of Life Sciences, College of Science and Art Mahyel Aseer, King Khalid University, Abha 62529, Saudi Arabia; snsir@kku.edu.sa; 10 Department of Biology, College of Science, King Khalid University, Abha 61421, Saudi Arabia

**Keywords:** biofertilizer, plant diseases, compost, nutrient transformation, PGPR

## Abstract

Microbial compost plays a crucial role in improving soil health, soil fertility, and plant biomass. These biofertilizers, based on microorganisms, offer numerous benefits such as enhanced nutrient acquisition (N, P, and K), production of hydrogen cyanide (HCN), and control of pathogens through induced systematic resistance. Additionally, they promote the production of phytohormones, siderophore, vitamins, protective enzymes, and antibiotics, further contributing to soil sustainability and optimal agricultural productivity. The escalating generation of organic waste from farm operations poses significant threats to the environment and soil fertility. Simultaneously, the excessive utilization of chemical fertilizers to achieve high crop yields results in detrimental impacts on soil structure and fertility. To address these challenges, a sustainable agriculture system that ensures enhanced soil fertility and minimal ecological impact is imperative. Microbial composts, developed by incorporating characterized plant-growth-promoting bacteria or fungal strains into compost derived from agricultural waste, offer a promising solution. These biofertilizers, with selected microbial strains capable of thriving in compost, offer an eco-friendly, cost-effective, and sustainable alternative for agricultural practices. In this review article, we explore the potential of microbial composts as a viable strategy for improving plant growth and environmental safety. By harnessing the benefits of microorganisms in compost, we can pave the way for sustainable agriculture and foster a healthier relationship between soil, plants, and the environment.

## 1. Introduction

In recent years, global food security has been increasingly threatened by the combination of population increase and the scarcity of limited arable land worldwide [1,2]. Consequently, the need to boost crop productivity has become a significant challenge in order to meet the demands of a continuously increasing global population [3]. To enhance crop productivity, various chemical fertilizers have been extensively employed worldwide; however, this widespread use has led to the deterioration of both human health and environmental ecology with significant severity [4,5]. Biofertilizers offer a promising solution in order to counteract the harms associated with chemical fertilizers. They are an essential component of integrated nutrient management, contributing significantly to crop output and food security in the agricultural sector [6,7]. Biofertilizers consist of one or more beneficial microbes capable of colonizing the interior or exterior of plants after soil application, as seeds, or as direct exposure on the plants. This colonization facilitates the enhancement of nutrient supply to the host plant, effectively promoting its growth [8,9]. These microbe-containing fertilizers improve the soil fertility through different mechanisms including atmospheric nitrogen fixation, solubilization of unavailable nutrients (such as phosphate, zinc, potassium, and iron), and synthesis of phytohormones [10]. Thus, the biofertilizers improve soil fertility and crop yield by harvesting the natural biological system of nutrients and recycling them [11]. The efficiency and efficacy of biofertilizers has remained controversial in terms of their application in the field and their potential to replace the chemical fertilizers, mainly due to the heterogeneous nature of soil, adaptability to harsh ecological conditions, and unsuitable formulations and carrier materials [12]. Thus, if a nutrient-rich carrier material with potential to facilitate microbial growth is used, outcomes from biofertilizers can be substantially enhanced and the use of chemical fertilizers can be completely or partially cut down. Nowadays, as a result of intensive agricultural activities, a huge amount of organic waste is generated by agricultural fields, and the disposal of this poses difficulties [13].

Production of nutrient-rich compost from agricultural waste has been emerging as an alternative way of disposal [14]. A dark brown or black earthy matter, rich in micro- and macronutrients and produced as a result of aerobic decomposition of biodegradable waste is known as compost [15]. Compost not only enhances the soil fertility in terms of micro- and macronutrients, it also improves the soil architecture by improving water- and air-holding capacity for better root growth. Moreover, compost can play an important role in the bioeconomy because its production does not depend on finite inputs [16]. This technique of waste disposal has proved to be very economic, as it enhances plant nutrient uptake, soil organic matter content, crop yield, and soil biophysical parameters [13,17]. The application of compost has been proven beneficial, as it has good impact on environment and soil quality [18].

Composting is the process in which organic materials like food scraps, leaves, twigs, lawn clippings, wood waste, and other organic matter are decayed by the soil microorganisms under controlled ambiance [15,19]. To carry out the decomposition process, either a pit can be dug in the ground, or the process can be conducted in special vessels called compost bins [20]. Certain factors, including temperature, proper aeration, pH, the quality and quantity of feedstock, etc., must be considered before initializing the process of composting to make it more efficient and reliable [15]. Another key factor to be considered is the ratio between carbon and nitrogen (C/N), which plays an essential function in the composting process. The application of compost enhances the physico-chemical properties of the soil and also exerts a positive impact on the soil’s microbial diversity [21]. The widespread adoption of compost in agriculture faces constraints due to its extended time of action and comparatively reduced nutrient supply to crops when compared with chemical fertilizers [17]. Various reports have been published on the composting of agricultural feedstock and its potential application in improving soil fertility and plant growth [22,23]. Moreover, the potential role of different phytobeneficial microbial communities to the process of composting has also been elucidated by several researchers around the world [24,25].

This review examines the feasibility and advantages of producing microbial compost-based biofertilizers using agricultural feedstock enriched with decomposing phytobeneficial microorganisms. Compost serves as an ideal carrier material for potential plant-beneficial microorganisms, presenting a possible alternative to chemical fertilizers. The application of microbial compost in fertilization holds immense promise for bolstering soil sustainability and elevating overall plant productivity. Additionally, we explore the factors influencing the microbial composting process and highlight the future prospects of adopting microbial compost-based biofertilizers.

## 2. Biofertilizers and Their Advantages

Agricultural productivity is decreasing continuously due to nutrient deficiency in the soils and the growth of obnoxious weeds and pests [26]. Conversely, the production cost has been raised over the last two decades. As a result of these factors, the growth rate in agriculture is falling behind the rapid pace of population growth [27,28]. The population explosion around the globe makes the use of chemicals fertilizers for improving crop production in order to meet food requirements inevitable [29]. Frequent soil tillage, chemical fertilizers, and narrow crop rotations are some of the intensive agricultural techniques that increase production in a short time. However, over time, these techniques have led to a decline in soil organic carbon, soil aggregation strength, and biodiversity, consequently reducing the productivity of field crops. Additionally, they contribute to air and groundwater pollution [30,31]. In this scenario, the biofertilizers have a great potential to improve soil fertility, thus reducing the need for the application of chemical fertilizers. Biofertilizer-mediated agricultural practices also result in crops with improved yield [32,33].

Biofertilizers utilize beneficial microorganisms to optimize plant growth by increasing nutrient supply, effectively enhancing overall nutrient availability, and promoting healthier and more robust plant development [34,35]. Generally, the potential of different beneficial microbes, including nitrogen fixers, phosphorus solubilizers, potassium solubilizers, iron mobilizers, as well as the microbes capable of producing phytohormones, is utilized during biofertilizer synthesis which improves the nutrient profile of the soil [36,37]. Subsequently, the microbe-oriented nutrient recycling through biofertilizers enhances the soil organic matter content and maintains the soil health and sustainability, which is ultimately followed by healthy plant growth. Several bacterial species, known as plant-growth-promoting rhizobacteria (PGPR), such as *Azotobacter* spp., *Escherichia coli*, *Pseudomonas* spp., and *Bacillus* spp., and arbuscular mycorrhizal fungi (AMF), such as *Glomus versiforme*, *Aspergillus awamori*, *Glomus macrocarpum,* and *Sclerocystis coremioides,* are often exploited in broad spectrum biofertilizers without causing any negative chemical influence on the soils [38,39,40]. Moreover, co-inoculation of PGPR and AMF also results in enhanced plant growth, nutrient uptake, disease tolerance, and resistance to abiotic stress. For example, co-culture of *Rhizobium*, *Azotobacter,* and vesicular arbuscular mycorrhiza (VAM) as biofertilizer enhanced the straw and grain yield when applied to wheat plants along with rock phosphate as a phosphate source [41]. Similarly, a mixed culture comprising *Thiobacillus thioxidans*, *Bacillus subtilis*, and *Saccharomyces* sp. was found capable of converting micronutrients into soluble forms such as Mn, Zn, Fe, etc., and making them available to plants [33]. *Trichoderma* spp. are also known to improve the tolerance of plants to biotic and abiotic stresses by producing enzymes that can detoxify harmful chemicals and by increasing the production of stress-response proteins in the plant [42]. For example, *Trichoderma harzianum* inoculation improved tomato plants’ tolerance to chilling stress by enhancing physiological, biochemical, and molecular responses [43]. The biofertilizers that achieve nitrogen fixing, i.e., potassium- and phosphate-solubilizing bacterial strains, significantly improve the growth, production, and qualitative characteristics of food crops [44]. Hence, these microbial-based fertilizers can significantly contribute to the establishment of sustainable agriculture systems, playing a pivotal role in achieving this goal.

Overall, the utilization of biofertilizers has been in practice for a considerable period due to their eco-friendly nature and superior cost-efficiency compared with chemical fertilizers. The biofertilizers can also be used to convert complex organic materials into simple compounds, followed by a change in the color and texture of the soil [45,46]. They have the capability to enhance the crop yield by 25–30% and can help the soil fight against dehydration and other soil-borne illnesses [15]. Therefore, to promote agricultural productivity, it is essential to acknowledge the application of biofertilizers [47,48].

## 3. Microbial Compost

Agricultural waste is now emerging as a compelling and cost-effective resource that can provide organic matter and essential plant nutrients to the soil, effectively supporting crop production [49]. However, raw organic waste cannot be directly applied because it is unsuitable for land and agricultural crops [50]. Hence, composting stands out as one of the most favorable, straightforward, and cost-effective methods employed to treat this type of wastes [51,52]. Compost can be locally produced on the farm and is an attractive technique of waste disposal. It is can recover valuable plant nutrients and improve the soil’s biophysical characteristics, soil organic matter, and crop yield [53,54]. As the nutrients in compost are slowly released depending upon the microbial biomass, the gradual nutrition availability for the plants is ensured [55]. Thus, the utilization of microbial compost aids in preventing nutrients leaching into groundwater and significantly increases soil productivity in the long term. The soil quality can be enhanced by repeated compost applications, which result in the enriching of microorganisms that are beneficial to the soil, an increased total carbon content, an enhanced cation exchange capacity, and a reduction in the abundance of plant-parasitic nematodes [56]. The use of pesticides and herbicides also decreases due to the improved plant resistance against diseases as a result of compost application [57]. The application of compost, along with certain soil microorganisms such as PGPR and AMF, has been proven to effectively boost soil fertility and health [58].

### 3.1. The Composting Process

**Figure 1:**
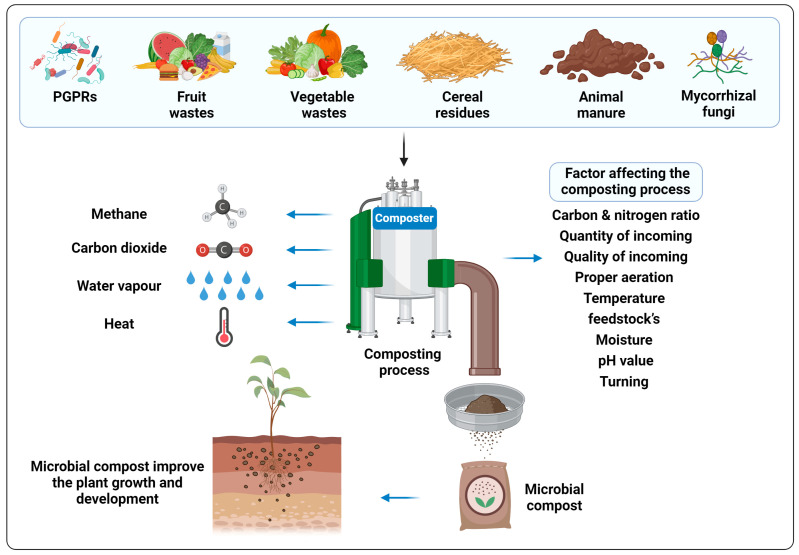
Schematic representation of the composting process.

The process of controlled decomposition of organic material like agricultural residues is known as composting [59]. A composting process requires the synergistic action of the natural forces that decompose the organic waste into the organic fertilizer to occur in a safe way [60]. The primary raw materials utilized for composting include agricultural waste such as fruit and vegetable waste, domestic kitchen residues, various crop residues (e.g., stover, cobs, and leaves), and different types of manure, e.g., cattle and poultry [61]. Both aerobic and anaerobic bacteria are involved in the composting process. For better a understanding of the degradation processes that occur in compost, characterization and identification of microorganisms is necessary in the composting process. Common PGPR, like *Azotobacter* spp., *Pseudomonas* spp., *Escherichia coli*, and *Bacillus* spp., and some AMF, such as *Aspergillus awamori*, *Glomus macrocarpum,* and *Sclerocystis coremioides,* were found to be involved in the decomposition process [62,63]. These microorganisms utilize amino acids, sugars, and lipids present in the feedstock as their energy source [64,65]. Composting offers numerous benefits beyond providing a significant release of various nutrient elements for plants. It contributes to soil conditioning, facilitates efficient manure handling, reduces the risks associated with different pollutants and weed seeds, and promotes pathogen destruction through a high-temperature composting processes [66]. A schematic diagram of the composting process has been presented in Figure 1.

### 3.2. The Biochemistry of Composting

The compost feedstock is often rich in organic compounds that are rich in components like carbon (C), hydrogen (H), oxygen (O_2_), and nitrogen [67]. The lignin, sugars, fats, cellulose, and proteins that are important components of agricultural raw material facilitate the decomposition of organic compounds during the process of composting. These components become progressively more oxidized and form molecules with more functional groups that have, however, a lower molecular weight during the aerobic decomposition [68]. The product obtained after the decomposition of organic matter is known as humus. In addition, the biodegradation occurs under both anaerobic and aerobic circumstances, but the composting process proceeds excellently under aerobic conditions [69]. Many microbes can perform their function in the absence of O_2_, but the process of anaerobic respiration is less energy-efficient and it utilizes chemical species like sulfate, carbon dioxide, nitrates, sulfur, and oxidized metal ions. Hence, to suppress the development of anaerobic conditions, artificial aerobic conditions should be introduced in the compost pile through pulling or pushing the air [70,71].

### 3.3. Microorganisms Involved in Plant Growth and the Composting Process

The composting process can be characterized as the biodegradation of organic waste into useful products under controlled ambiance. These products are applied to the soil, and they effectively enhance the physico-chemical properties of the soil [72,73]. The biodegradation of organic waste is accelerated by diverse microbial species belonging to different microbial groups, namely, bacteria, actinomycetes, and filamentous fungi. The bacterial species belonging to the genera *Pseudomonas*, *Bacillus*, *Paenibacilus*, and *Enterobacter* were found to be the most abundant microorganisms, having a very high population density of 3.0 × 10^8^ CFU/g throughout the composting process, followed by actinomycetes (mainly the species of genus *Streptomyces*, *Nocardia*, *Micromonospora*, *Thermomonospora Dactylosporangium,* and *Kibdelosporangium*). The members of filamentous fungi that drive the composting process mainly belong to the genus *Aspergillus* and have a low population density (i.e., 1.2–1.6 × 10^8^ CFU/g) [72].

A group of specific microorganisms which positively influences plant growth are PGPR and AMF [48,74]. A number of PGPR and AMF, along with their potential roles in plant improvement, are presented in Table 1. These microbes belong to many genera, including *Xanthomonas*, *Agrobacterium*, *Streptomyces*, *Alcaligenes*, *Cellulomonas*, *Arthrobacter*, *Amorpho sporangium*, *Bacillus*, *Pseudomonas* sp., *Azotobacter*, *Actinoplanes*, *Rhizobium*, *Erwinia*, *Bradyrhizobium*, *Enterobacter*, *Rhizophagus irregularis*, and *Glomus intraradices* [6]. Generally, the microorganisms that are used as BF are potassium solubilizers, phosphorus solubilizers, nitrogen fixers, and phytohormone producers [75]. Nitrogen-fixing microbes convert atmospheric N_2_ to ammonia [76]. Some microorganisms belonging to the *Ectorhizospheric* strains and *Endosymbiotic rhizobia* have been defined as efficient phosphate solubilizers [7,77]. The most potent strains from bacterial genera that solubilize phosphorus are *Pseudomonas*, *Bacillus*, *Enterobacter*, and *Rhizobium* genera [9,78]. Similarly, numerous PGPR species belonging to different genera, namely, *Bradyrhizobium*, *Pseudomonas*, *Rhizobium*, *Agrobacterium*, *Klebsiella*, *Enterobacter*, *Azotobacter*, and *Bacillus,* are best known for their phytohormones production potential. These microbe-oriented phytohormones have an effective role in plant growth stimulation.

**Figure 2:**
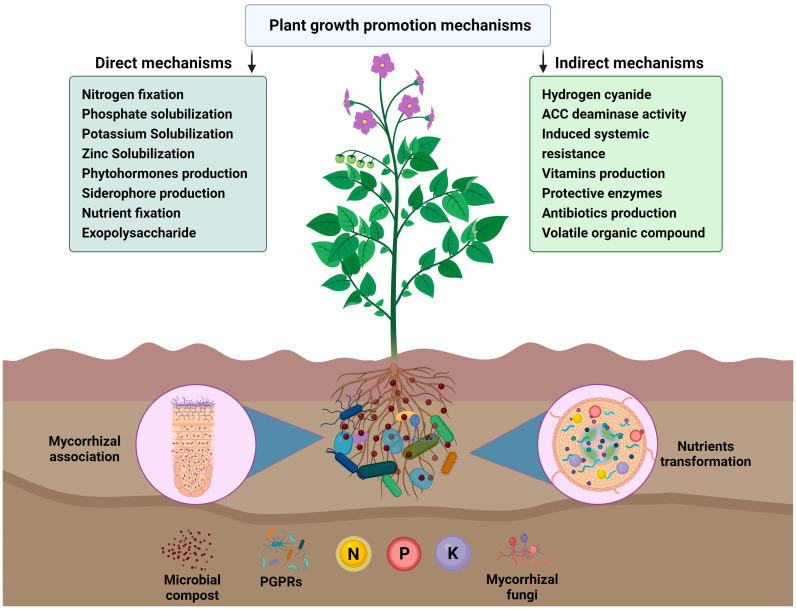
Schematic representation of the compost-based biofertilizers. Mycorrhizal fungal filaments and plant-growth-promoting rhizobacteria in the soil act as to support the development of the plant root system and are more effective at water and nutrient absorption than the roots themselves. PGPR and AMF also explore the soil and reach places unattainable to roots and increase nutrient uptake by plants from the soil. Their effects on plant growth through two different mechanisms, such as direct mechanism and indirect mechanism, are illustrated.

The ability of PGPR to solubilize potassium rock by secreting organic acids has also been investigated. A number of bacterial strains, including *Burkholderia* sp., *Bacillus mucilaginosus*, *Ferrooxidans* sp., *Paenibacillus* sp., *Pseudomonas* sp., *Bacillus edaphicus*, and *Acidothiobacillus* sp., are PGPR that solubilize potassium-bearing minerals, thereby releasing the potassium in the form that is available for plants [79]. Therefore, utilizing PGPR as biofertilizers to enhance agriculture can reduce the dependence on agrochemicals and promote sustainable crop production (Figure 2).

**Table 1:** Some PGPR and AMF strains involved in plant growth promotion.

Chemicals	Microorganisms	Beneficial Effects	References
**Direct Mechanism**			
Nitrogen fixation	*Bradyrhizobium japonicum*, *Glomus macrocarpum*, *Azotobacter vinelandii*, *Bacillus*, *Rhizobium*, *Beijerinckiaderxii*, *Klebsiella pneumoniae*, *Enterobacter cloacae*, *Citrobacterfreundii,* and *Pseudomonas putida*	The conversion of atmospheric N_2_ into plant-utilizable forms triggers improvement in plant development and yield	[80,81,82,83,84,85]
Phosphate solubilization	*Penicillium brevicompactum*, *Aspergillus niger*, *Pseudomonas striata*, *Enterobacter*, *Erwinia*, *Bacillusmegaterium*, *Ochrobactrumanthropi*, *Bacilus*, *Beijerinckia*, *Burkholderia*, *Rhizobium,* and *Serratia*	Solubilizing the inorganic phosphorus from insoluble compounds and making them available to the plants	[86,87,88,89,90]
Potassium solubilization	*Aspergillus niger*, *Aspergillus terreus*, *Acidothiobacillus* sp., *Bacillus edaphicus*, *Ferrooxidans* sp., *Bacillus mucilaginosus*, *Pseudomonas* sp., *Burkholderia* sp., and *Paenibacillus* sp.	Solubilizing potassium rock by producing and secreting organic acids and making them available to the plants for growth and development	[79,91,92,93]
Zinc mobilization	*Beauveria caledonica*, *Hymenoscyphus ericae*, *Oidiodendron maius Pennisetum glaucum*, *Gluconacetobacter diazotrophicus*, *fluorescent pseudomonads*, and *Bacillus* sp.	Solubilizing the insoluble Zn into soluble form and hence having efficient role in plant growth and development	[94,95,96,97,98,99]
Production of phytohormones	*Paecilomyces formosus*, *Asprgillus fumigatus*, *Fusarium proliferatum Azotobacter*, *Arthrobacter*, *Azospirillum*, *Pseudomonas*, *Bacillus*, *Acinetobacter*, *Flavobacterium*, *Enterobacter*, *Micrococcus*, *Agrobacterium*, *Clostridium*, *Rhizobium*, and *Xanthomonas*	Play an important role as regulators of growth and development of plants	[100,101,102,103,104,105]
Siderophore production	*Aspergillus fumigatus*, *Glomus etunicatum*, *Glomus mossae*, *Trichoderma* spp., *Pseudomonas fluorescens*, *Rhodococcus*, *Acinetobacte*, and *Pseudomonas putida*	Solubilize and sequester iron from the soil and then provide it to the plant cells	[86,106,107,108,109]
Exopolysaccharides production	*Azotobacter vinelandii*, *Bacillus drentensis*, *Enterobacter cloacae*, *Rhizobium* sp., *Agrobacterium* sp., and *Xanthomonas* sp.	Plays a pivotal role in increasing the number of soil macropores, aggregating rhizospheric soil particles, and maintaining water potential	[110,111]
**Indirect Mechanisms**			
Hydrogen cyanide	*Alcaligenes*, *Aeromonas*, *Rhizobium*, *Pseudomonas,* and *Bacillus* sp.	Powerful inhibitor of many metal enzymes, especially copper-containing cytochrome Coxidases	[112,113,114]
ACC deaminase activity	*Gigaspora rosea*, *Achromobacter*, *Azospirillum*, *Pseudomonas*, *Enterobacter*, *Bacillus*, and *Rhizobium*	Plants were able to tolerate environmental stresses by keeping a normal amount of ethylene in their root zone	[115,116,117]
Induced systemic resistance	*Trichoderma virens*, *Pseudomonas,* and *Bacillus* spp.	Induced resistance is the state of an enhanced defensive ability developed by plants when appropriately stimulated	[118,119,120,121]
Production of vitamins	*Glomus aggregatum*, *Glomus viscosum*, *Azotobacter vinelandii*, *Azospirillum brasilense*, *Azospirillum* spp., *Pseudomonas fluorescens*, *Rhizobium leguminosarum*, *Rhizobium etli*, *Sinorhizobium meliloti*, *Mesorhizobium loti*, and *Bacillus subtilis*	Facilitate the production of essential compounds for plants and bacteria, induce resistance against pathogens, and directly promote plant growth	[122,123,124]
Production of protective enzymes	*Aspergillus niger*, *Glomus* spp., *Bacillus*, *Burkholderia*, *Enterobacter*, *Pseudomonas*, *Serratia,* and *Staphylococcus*	May have a dramatic effect on the cycling of nutrients such as phosphorus, nitrogen, and sulfur	[125,126,127]
Production of antibiotics	*Pseudomonas*, *Bacillus,* and *Azotobacter*	Prevent the detrimental effects of pathogens on plants through production of inhibitory substances	[128,129]
Volatile organic compound (VOCs)	*Bacillus amyloliquefaciens*, *Bacillus subtilis*, *Pseudomonas fluorescens*, *Bacillus mojavensis*, *Trichoderma* spp., *Trametes gibbosa*, and *Trametes versicolor*	VOCs have a significant role in the plant growth promotion and suppression of plant diseases	[130,131,132,133]

## 4. The Formulation of the Microbial Compost-Based Biofertilizers

Enriching compost with nutrients, beneficial bacteria, and AMF is a key strategy for improving its nutritional value and enhancing its positive effects on plant growth [134]. By establishing a mutualistic relationship, soil microorganisms and plants work together to facilitate nutrient uptake without disrupting the overall physico-chemical or biological equilibrium of the system. Endophytic PGPR, for example, resides within the plant roots, positively impacting the plants through the secretion of phytohormones, nitrogen fixation, enhanced phosphorus uptake, and the solubilization of inorganic phosphates. This cooperative partnership promotes healthier plant growth and fosters a sustainable ecosystem [135,136]. In general, supplementation of compost with different types of nutrients as well as its bioaugmentation with potential PGPR and AMF strains can lead to value addition in BF technology. Thus, such compost-based biofertilizers need to be added in future long-term farming strategies in order to make the farm yield more sustainable and cost-effective. For this purpose, the compost can be developed through natural processes followed by adding the inoculum of known PGPR or AMF strain(s) at an optimal population density. However, the survival of PGPR or AMF strain(s) in the compost and their synergistic effect, along with natural microflora of compost, are vital factors, which need to be assessed before developing compost-based biofertilizers.

## 5. Feedstock for Microbial Compost-Based Biofertilizers

The number of global agricultural products is rising steadily despite the global decrease in cultivated land area, due to the increased demand to produce food to feed an increasing population [137,138]. The United Nation’s Food and Agriculture Organization (FAO) predicted that the need for food will rise rapidly as the population of the world will surpass eight billion by 2030 [139]. Agricultural waste and residue will also increase as the food production increases [140]. The physiochemical properties and composition of the final compost are mainly dependent on the agricultural residues or waste which are used as a feedstock. Many essential characteristics of the compost, including the C-to-N ratio, available micronutrients (e.g., Fe, Mg, Mo) and macronutrients (e.g., N, P, K, Ca) for the plants, as well as the texture and structure of the compost, depend on the method of composting and the type of feedstock used [66]. The feedstock used in composting varies remarkably from area to area and season to season depending on the type of agricultural produce. Various classes of agriculture feedstocks being used for composting include cereal residues, animal manure, fruit and vegetable waste, and grassland residues [140,141].

### 5.1. Cereal and Grassland Residues

Cereal residues encompass the materials remaining in croplands after crop harvest, comprising stems, leaves, straw, and seedpods. Additionally, process residues like husk, roots, bagasse, and seeds are the by-products remaining after the processing of cereal crops [142]. The harvesting of cereals generates a substantial amount of agricultural residues, which are frequently underutilized as livestock feedstock or for other purposes. For instance, straw is one of the primary agricultural residues left after harvesting several cereal crops [143]. Due to its low nutritional value, straw is not considered a suitable livestock feedstock, leading to the common practice of leaving it in the fields to enhance the soil’s carbon stock. Alternatively, straw is used for livestock housing and bedding, as well as in various horticultural applications [144]. Despite being used for livestock housing, straw requires periodic disposal. However, it has been found to be a suitable substrate for anaerobic digestion. To achieve the highest compost yields, mechanical, thermochemical, or biological pre-treatment of the straw is necessary to reduce digestion retention times compared with other substrates. Major agricultural residues like rice straw, wheat straw, corn stover, and sugarcane bagasse are abundant and readily available for compost production, offering valuable opportunities for sustainable waste management and resource utilization in agricultural systems [145].

About 70% of the world’s agricultural land and 26% of the world’s total land area is comprised of grasslands [146,147]. In terms of purpose and harvesting type, grassland is usually classified as permanent grassland and temporary grassland. The European agricultural sector is very much dependent on temporary grasslands; they are the most significant origin of feed for livestock production. Since the use of grass silage as animal feed is decreasing, grass is receiving more attention as an alternative feedstock for composting [61].

### 5.2. Fruit and Vegetable Waste

Fruits and vegetables are essential natural sources of minerals, dietary fibers, and vitamins in both developing and developed countries. The fruit and vegetable production is continuously improving in many developing countries in, for example, South America and Asia [148]. The largest source of vegetables, accounting for more than 61% of the global output, is Asia. Similarly, according to an estimate, about 30% of the world’s fruit is supplied by India, Brazil, and China [149]. Items that are extracted from vegetables and fruits during processing, cleaning, cooking, and packaging are considered vegetable and fruit solid waste. Many of these by-products are either abandoned or landfilled. The major fruit and vegetable waste products which can be used for preparing compost include twigs, pomace, skins, rinds, cores, pulp, stems, pits, leaves, seeds, peels, and spoiled fruits and vegetables [150].

### 5.3. Animal Manure

Animal manure and slurry cause environmental pollution due to the improper recycling of them [151]. The composting of animal manure is an efficient strategy to manage the environmental risks related to animal manure. The composting process converts the animal manure into high-quality products that can be utilized for numerous agricultural purposes [152]. Various factors like low porosity, high humidity, low C/N ratio, and high pH affect the manure composting process. Thus, for the production of quality compost, efficient management techniques for manure composting is a necessary pre-requisite. In order to obtain a quality product in less time and at a lower cost, various different aeration techniques, bulking agents, substrate-conditioning feed stocks, and other available options have been used for the composting of manure [153,154]. The bulking agent added during composting of animal manure improves the mineralization rate of the substrate by optimizing its properties like porosity, humidity content, pH, particle density, C/N ratio, etc. Generally, for the composting of nitrogen-rich waste (such as animal manure), agricultural and forestry by-products are used as bulking agents. The most commonly used materials are cotton waste, hay, and cereal straw [155]. The high carbon content of these bulking agents increases the low C/N value of animal manures [156].

## 6. Plant Growth Promotions Mediated by Microbial Compost-Based Biofertilizers

Microorganisms are an integral part of soil nutrient cycling in soil–plant–microbe systems. Plant growth and development is facilitated by the soil microbial community in several different ways [157]. Generally, microorganisms promote plant growth by improving nutrient mobilization and availability through a number of mechanisms such as nitrogen fixation, organic compounds mineralization, mineral nutrients solubilization, phytohormone production, etc. [158,159]. Some rhizospheric microbes improve plants’ physiology and resistance against phytopathogens indirectly by producing substances that act synergistically with the native immune system of a plant to neutralize the harmful effects of pathogens [160,161]. Through several activities, the microorganisms either enhance the growth of plants or suppress the disease. The major microbial direct and indirect mechanisms involved in plant growth promotion are presented in Table 2 and briefly described below.

### 6.1. Direct Mechanisms of Microorganisms’ Influence on Plants

#### 6.1.1. Nitrogen Fixation

Nitrogen (N) is an essential plant nutrient. Significant energy is needed to break the triple bonds between the two N-atoms of atmospheric molecular nitrogen (N_2_), because the plants are unable to use the atmospheric molecular nitrogen (N_2_) directly [205,206]. Plants normally use inorganic forms of nitrogen like NO^3−^ and NH^4+^ as well as low-molecular-weight dissolved organic nitrogen (DON), especially amino acids. Plants cannot utilize the atmospheric dinitrogen (N_2_) unless some diazotrophic microorganisms reduce it to more usable form like ammonia (NH_3_) [207]. In biological nitrogen fixation (BNF), nitrogenase enzyme present in diazotrophic microorganisms catalyzes and converts the atmospheric N_2_ to NH_3_. BNF provides more than 2 × 10^13^ g nitrogen annually throughout the world. Symbiotic fixation adds about 80% of this amount, while the remaining is contributed by either free-living or associative nitrogen-fixing systems [208,209]. Bacteria and fungi are the only organisms capable of converting atmospheric N2 into a form that plants can utilize. Symbiotic and associative symbiotic interactions with plant roots are accomplished by different AMF species including *Glomus macrocarpum*, *Glomus hoi,* and *Glomus mosseae* [82,83,84] and several bacterial species like *Pseudomonas* spp., *Alcaligenes* spp., *Azotobacter* spp., *Azospirillum* spp., *Enterobacter* spp., *Arthrobacter* spp., *Acinetobacter* spp., *Bacillus* spp., *Bradyrhizobium japonicum*, *Serratia* (*Brady*) *rhizobium* spp., and *Burkholderia* spp. [210,211]. Compost consisting of nitrogen-fixing microbial strains showed improved nitrogen metabolism. It has been reported that the species of genus *Gordonia* exhibits nitrogenase reductase activity and was found to be involved in the nitrogen metabolic reaction of compost [212]. Thus, adding nitrogen-fixing PGPR to compost can enhance the growth of plants by improving the nitrogen availability to plants.

#### 6.1.2. Phosphate Solubilization

Phosphorus (P) is the second most crucial nutrient for plant growth, and its scarcity can severely limit crop production to a critical level [213]. To address P deficiency in soils, agricultural fields are commonly treated with various types of chemical fertilizers. However, a major portion of P fertilizers is fixed in soils and becomes unavailable for plants. For example, tropical and subtropical soils come under the classification of phosphorous-deficient soils due to their high pH [214]. One of the economical ways to deal with P deficiency in soils is to focus on rock phosphate sources in the soil and make them available for plants. Various microorganisms have the ability to solubilize fixed forms of P such as rock phosphate. Rhizobacterial species reported to solubilize phosphorous include *B. megatheriumand*, *Enterobacter*, *Erwinia*, *O. anthropi* TRS-2, and *Pseudomonas striata* [86]. Such bacteria make the soil rich with soluble inorganic phosphate by decomposing phosphate-rich organic compounds. *Enterobacter*, *Serratia*, *Azotobacter*, *Beijerinckia*, *Bacillus*, *Pseudomonas*, *Burkholderia*, *Microbacterium*, *Erwinia*, *Rhizobium*, and *Flavobacterium* are amongst the most important bacterial genera that have been reported to solubilize phosphate [36]. Rivera-Cruz et al. (2008) revealed that mixing of P-solubilizing microbial strains with compost improved plant physiology and the physical and microbiological characters of the soil [215]. These modified biofertilizers have been reported to enhance the available P content of the soil [216,217].

#### 6.1.3. Potassium Mobilization

Potassium (K) is an essential macronutrient and is required for plant growth. The concentration of soluble potassium is usually low in soil because more than 90 percent of the potassium exists in the form of silicate minerals and insoluble rock. Potassium deficiency is also one of the major constraints for an optimal crop production [218,219]. The plants suffering from potassium deficiency often suffer from poor root development, low seed production, slower growth, and smaller yields [220]. In order to cope with potassium deficiency without using mineral fertilizers, one of the possible ways is to focus on the alternative endemic sources of potassium present in unavailable rock forms in the soils [221]. Several soil microbes have been isolated and characterized for their potential to solubilize potassium rock and to make it available to plants through the production and secretion of organic acids. Several PGPR, including *Paenibacillus* sp., *Pseudomonas* sp., *Bacillus edaphicus*, *Acidothiobacillus* sp., *Burkholderia* sp., *Bacillus mucilaginosus*, and *Ferrooxidans* sp., have been reported to release potassium in an accessible form by solubilizing potassium-bearing minerals in soils [79]. Similarly, AMF such as *Aspergillus terreus* and *Aspergillus niger* have demonstrated the capability to solubilize potassium and produce acids by utilizing feldspar and potassium aluminum silicate as insoluble K sources [93]. Hence, by exploiting the potential of potassium-solubilizing microorganisms, an eco-friendly crop production can be supported by reducing the use of agrochemicals. Grigiz MGZ (2006) concluded that the combination of compost and K-mobilizing bacterial strains (*Bacillus* sp. UBFBa4 and UBFBa7) improves the growth of wheat by increasing the uptake of K. The compost generally caused an increase in the population density of K-mobilizers, which convert the insoluble K into plant-useable forms by lowering the pH through organic acid production [222].

#### 6.1.4. Zinc Solubilization

Zinc (Zn) is an important nutrient required by the plant tissues in small amounts for growth and gene regulation [223]. Increasing the zinc concentration in crop plants is a major challenge However, due to the relatively low solubility and decreased availability of Zn in agricultural soils, there is a prevailing universal Zn deficiency in crops [223]. A prediction reveals that about 50% of the total Asian agricultural soils are deficient in zinc [224]. The deficiency of Zn is because of several reasons depending on the soil condition; for example, the solubility of Zn reduces with an increase in pH, high magnesium-to-calcium ratio, high availability of P and Fe, and variability in organic matter and bicarbonate content [97,225]. Using Zn fertilizers in the fields can overcome Zn deficiency, but generally, chemical fertilizers are expensive and have unfavorable effects on the environment. Thus, there should be an eco-friendly and cost-effective approach to overcoming the Zn deficiency. Thus, PGPR like *Bacillus* sp., *Gluconacetobacter diazotrophicus*, *fluorescent pseudomonads,* and *Pennisetum* have been reported to increase Zn solubility in soil [96,97,98,99]. Similarly, some AMF like *Beauveria caledonica*, *Hymenoscyphus ericae,* and *Oidiodendron maius* have been found to be Zn solubilizers [94,95]. Compost combined with Zn-solubilizing microbial strains was found to improve the crop productivity by increasing Zn mineralization and uptake in plants. Shanmugam PM (2000) reported that the application of green manure compost amended with Zn-solubilizing PGPR enhances the growth and physiology of rice plants. Thus, modification of compost by inoculating Zn-solubilizing microorganisms is a viable strategy for improving the bioavailability of this micronutrient to plants to achieve sustainable agricultural production [226].

#### 6.1.5. Production of Phytohormones

Plant growth hormones are organic compounds utilized by plants as messengers to interact with and respond to their environment [227]. These hormones are very efficient if manufactured in a minute quantity; however, they may result in plant growth inhibition if found in a greater amount [228]. These plant growth hormones can elicit physiological actions that influence growth and fruit ripening, as they are produced in one part of the plant and transported to other parts [229]. In addition to plants, several PGPR and AMF have demonstrated the ability to produce growth hormones, i.e., *Azospirillum*, *Enterobacter*, *Pseudomonas*, *Bacillus*, *Azotobacter*, *Paecilomyces formosus*, *Asprgillus fumigatus,* and *Fusarium proliferatum*. Amongst the phytohormones, indole-3-acitic acid (IAA) plays a significant role in the growth and division of cells and increases the growth of lateral roots in plants [103,104,228,230]. IAA functions as a signal molecule in plant development, contributing to processes such as organogenesis. Moreover, it plays a crucial role in gene regulation and cell division, elongation, and expansion. Phytohormones are manufactured by the bacteria that solubilize phosphate [231,232]. Various bacterial strains with the potential to produce IAA in pure cultures and soils, along with their interactions with plants roots, have been widely studied [230,233].

IAA has a role in organogenesis, gene regulation, cell division, cell elongation, and cell expansion [234,235]. It is required not only for phytostimulation but is also used by various microorganisms for their interaction with plants due to its significance in bacterial colonization of plant roots. It directly influences bacterial physiology, as it functions as a signaling molecule for bacteria as well [236]. Sometimes, IAA production by the microbial strains is triggered in the presence of tryptophan, which serves as a precurser for its production [237]. For example, a free-living nitrogen-fixing bacterium, *Azospirillum*, is distinguished in terms of its production of a significant concentration of IAA to stimulate plant growth [238]. Active IAA-producing PGPR are often isolated and characterized from the plant rhizospheres and re-inoculated with plants for plant benefits [239]. For example, when eucalyptus cuttings were grown on a substrate that was inoculated with IAA-producing rhizobacteria, a notable development in root proliferation and root dry matter was observed [240]. It has been documented that the integrated use of organic manure compost and phytohormones-producing biofertilizers improves the growth parameters (such as leaf number and plant height) of onions [241]. Both compost and biofertilizers synergistically produce plant growth regulators (i.e., IAA and gibberellins) and influence the plant growth in more favorable ways.

#### 6.1.6. Siderophore Production

Siderophores are low-molecular-weight substances which have chelation properties and are primarily produced and employed by microorganisms to fulfil the nutritional requirements for iron [242]. Siderophores have the ability to form complexes with ferric ion through their ligands, improve its solubilization, and enable its removal from natural complexes or from minerals [243]. Iron hydroxide, being poorly soluble, remains unavailable to biological systems and, at physiological pH under aerobic conditions, the unstable ferrous (Fe^2+^) form of iron is oxidized and converted into ferric (Fe^3+^), which is a relatively more stable form [244,245]. When siderophores are released into an environment, they solubilize the iron by forming a ferric–siderophore complex, which can move through the diffusion process and reach the cell surface [246]. After the ferric–siderophore complexes are formed, different Gram-positive and Gram-negative bacteria recognize these complexes, and their transport systems start working. Several PGPR and AMF, including *Pseudomonas fluorescens*, *Rhodococcus*, *Acinetobacte*, *Pseudomonas putida*, *Aspergillus fumigatus*, *Glomus etunicatum*, *Glomus mossae*, *Trichoderma* spp., and *Aspergillus niger,* have been reported to harbor the potential for production of siderophores [86,106,107,108]. Microbial siderophores are categorized into four major classes based on the types of ligands and fundamental features of functional groups involved in iron complexation. These classes include carboxylates, pyoverdines, phenol catecholates, and hydroxamates [247]. The application of biofertilizers in 50 and 75% compost enhances the plant growth significantly. The biofertilizers added to compost exhibit siderophore-producing activity along with other plant-growth-promoting characters [248]. The compost with siderophore-producing microbial strains has a better ability to facilitate plant Fe uptake and also increase the microbial density of antagonistic bacteria involved in plant growth promotion.

#### 6.1.7. Exopolysaccharides Production

Exopolysaccharides (EPSs) are biodegradable polymers usually with a high molecular weight. Various bacteria, algae, and plants have been reported to produce these extrapolymeric substances [249]. EPSs play a pivotal role in increasing the number of soil macropores, aggregating rhizospheric soil particles, and maintaining water potential, thus helping plants ameliorate different stress conditions including salinity, drought, or water logging [250]. The EPS-producing rhizobacterial species include *Bacillus drentensis*, *Azotobacter vinelandii*, *Enterobacter cloacae*, *Rhizobium* sp., *Agrobacterium* sp., and *Xanthomonas* sp. [251]. The combination of compost and EPS-producing PGPR can improve soil fertility by improving soil porosity and structure and can also provide plants with the ability to tolerate various stress conditions.

### 6.2. Indirect Mechanisms of Microorganisms’ Influence on Plants

#### 6.2.1. Hydrogen Cyanide Production

Hydrogen cyanide (HCN) is a poisonous chemical which is produced by the soil microbes and has a low molecular weight and antifungal properties [112]. HCN is produced through the interaction of glycine with the HCN synthetase enzyme, which is located on the plasma membrane of specific rhizobacteria [252]. Until now, many microbial genera such as *Rhizobium*, *Aeromonas, Alcaligenes,* etc., have been reported to harbor the potential for HCN production in the rhizospheric soil and plant root nodules [253]. Many studies have revealed the disease-controlling effects of HCN, e.g., inhibition of “root-knot” and black rot in tomato and tobacco [254,255]. However, a few studies have also indicated the harmful effects of HCN on the metabolism and growth of root cells in different plants. For example, HCN produced by *Pseudomonas* in the rhizosphere was found to hinder the primary growth of roots in *Arabidopsis* because of the suppression of an auxin-responsive gene [255,256]. The use of HCN-synthesizing PGPR strains in conjunction with compost can be a viable approach to protecting plants from soil-borne phytopathogens and to improving plant growth and physiology with greater efficiency.

#### 6.2.2. ACC Deaminase Production

Plants require a suitable quantity of ethylene for their growth and development. However, its high concentration can influence the plant cellular processes and can hinder growth [257]. The level of ethylene in plants is also regulated by the soil microbes. For example, the ethylene concentration in the root region of *Arabidopsis thaliana* was found to be managed by soil microbes using their 1-amino-cyclopropane-1-carboxylic acid (ACC) deaminase, which stops ACC from acting in ethylene biosynthesis pathways [258]. Some PGPR and AMF strains, including *Azospirillum*, *Enterobacter*, *Bacillus*, *Achromobacter*, *Rhizobium*, *Pseudomonas,* and *Gigaspora rosea,* have the potential to produce ACC deaminase, an enzyme responsible for the cleavage of ACC into ammonia and α-ketobutyrate [115,116,117]. For example, Ghosh et al. (2003) achieved a higher root length in *Brassica* *compestris* through inoculation with three *bacillus* spp. (*Bacillus circulans* DUC1, *Bacillus firmus* DUC2, and *Bacillus globisporus* DUC3) carrying ACC deaminase activity. In another study, the shoot dry biomass was enhanced in *Brassica napus* after the inoculation with *Pseudomonas asplenii* harboring the ACC deaminase gene [259]. Therefore, PGPR with ACC deaminase activity boost plant biomass in a stressed environment like pathogenicity, high temperature, contaminants drought, salinity, waterlogging, etc. [260]. Plants showed an increase in root length and biomass when treated with compost containing ACC deaminase, producing plant-beneficial bacteria [261].

#### 6.2.3. Induced Systemic Resistance

Many non-pathogenic rhizobacteria and beneficial fungi have the ability to overcome diseases by producing a defense mechanism known as “Induced Systemic Resistance” (ISR) [262]. ISR is a state of increased basal resistance of the plant, which relies upon two signaling molecules (i.e., salicylic acid and jasmonic acid) [263]. Pathogens show variability in their sensitivity against signaling molecules that elicit resistance. The same strain shows antagonism against many phytopathogens. *Bacillus* spp. and *Pseudomonas* are the most examined rhizobacteria that activate ISR [119,264]. The treatment of crop plants with resistance-inducing and antagonistic PGPR and AMF leads to more effective biocontrol strategies for making the cropping systems better [264]. Several PGPR and AMF, such as *Trichoderma virens*, *Pseudomonas,* and *Bacillus* spp., have been used to induce systemic resistance in plants [120,121,265]. Thus, the fortification of compost by using ISR-inducing microbes can suppress numerous diseases in plants caused by soil-inhabiting pathogens. This blending of antagonistic microbes with compost could be an efficient strategy for improving plants’ basal resistance response against harmful microbial communities.

#### 6.2.4. Production of Vitamins

Microbes that produce vitamins are widespread in the rhizosphere. Vitamins are either obtained from root exudates or synthesized from rhizospheric bacteria and fungi [266,267]. In general, the vitamins secreted by plant roots are not exclusively synthesized by the plants themselves. Instead, they are produced by microorganisms and then taken up by the plants. Subsequently, the plants release these vitamins along with root exudates [123]. The composition of many vitamins like biotin, pyrroloquinoline quinone, niacin, thiamine, and pantothenic acid plays an important role in microbial chemotaxis [268]. Due to the frequent presence of microbial-produced vitamins in the rhizosphere and the adaptation of plants to be able to uptake microbially-synthesized vitamins, such vitamins are considered to play an important role in rhizosphere interactions [123,124]. The synthesis of thiamine and many other vitamins has been reported to be caused by numerous potential microbes such as *Glomus aggregatum*, *Glomus viscosum*, *Azospirillum brasilense*, *Rhizobium leguminosarum*, *Azotobacter vinelandii*, *Azospirillum* spp., *Bacillus subtilis*, *Sinorhizobium meliloti*, *Rhizobium etli*, *Mesorhizobium loti,* and *Pseudomonas fluorescens* [122,123,124]. The primary function of vitamins includes the induction of defenses against pathogens, being a cofactor in different metabolic pathways, the promotion of plant growth, and the production of essential compounds for plant growth [118]. The microbes harboring the potential to synthesize vitamins and compost may promote the proliferation and colonization of plant-beneficial microbes in the root zone that will subsequently enhance plant growth through the aforementioned mechanisms.

#### 6.2.5. Production of Protective Enzymes

Plant microorganisms possess the capacity to produce certain proteins that promote and enhance plant development and growth. Root symbionts, such as ectomycorrhizal fungi and other rhizosphere microorganisms, play a vital role in facilitating these processes [269]. The enzymes are utilized for defense mechanisms, but some play role in cell wall degradation, like hydrolytic enzymes. The synthesis of enzymes comprises cellulase, chitinase, protease, b-1, 3-glucanase, and lipase, but some fungal cells may be lost during their production [270]. Phytase, peroxidase, phenylalanine ammonia-lyase, and polyphenol oxidase are examples of the most exceptional defense enzymes [271]. Many PGPR and AMF can produce phytase enzymes that have the ability to mineralize the phytates, including beneficial species that produce protective enzymes, which belong to the *Burkholderia*, *Staphylococcus*, *Enterobacter*, *Bacillus*, *Pseudomonas* and *Serratia*, *Aspergillus niger,* and *Glomus* spp. [125,255]. In the rhizosphere, phosphatases, arylsulfatases, proteases, and other extracellular enzymes tend to exhibit higher activity compared with the bulk soil. This heightened enzymatic activity significantly impacts the cycling of nutrients, such as phosphorus, nitrogen, and sulfur [272]. *Pseudomonas*, *Bacillus*, and *Serratia* can provide numerous extracellular lytic enzymes including b-1, 3 glucanases, chitinases, cellulases, laminarinase, and proteases [273]. Protective enzymes producing microbes when mixed with compost may increase the soluble sugar level for plant uptake and utilization along with other nutrients (i.e., N and P). Thus, such modified forms of compost will help plants grow in stressed environments.

#### 6.2.6. Production of Antibiotics

The most significant antagonistic activity for combating phytopathogens is the generation of diverse antibiotics by microbes [274]. Several PGPR have been reported to produce different types of antibiotics including oligomycin A, viscosinamide, xanthobaccin, pyoluteorin, pyrrolnitrin, and 2,4 diacetyl phloroglucinol (2,4-DAPG) [255]. *P. fluorescens* BL915 strains produce the pyrrolnitrin that can stop the destruction of *Rhizoctonia solani* during the damping off of the cotton plant [275]. *Pseudomonads* can synthesize the 2,4-Dthat, which is a very active and extensively studied antibiotic, and can cause damage to the membrane of *Pythium* spp. [276]. Moreover, *Pseudomonads* produce the phenazine exhibiting redox activity, through which it can suppress the pathogens of plants such as *Gaeumannomyces graminis* and *F. oxysporum* [262]. The antibiotics generated by the majority of *Bacillus* spp. (circulin, colistin, and polymyxin) are very effective against Gram-negative and Gram-positive bacteria, and against many other pathogenic fungi as well [277]. In another study, different bacterial strains such as *Pseudomonas*, *Bacillus,* and *Azotobacter* exhibited a broad-spectrum antifungal activity on Muller–Hinton medium against different fungal pathogens such as *Aspergillus*, *Fusarium,* and *Rhizoctonia bataticola* [278]. Compost and antibiotic-producing bacteria can synergistically relieve plants from devastating microbes. Compost may facilitate the growth of beneficial microorganisms, which, in turn promote plant growth by retarding the growth of plant pathogens via their antimicrobial activity.

#### 6.2.7. Volatile Organic Compounds

Soil microorganisms generate different types of metabolites during their metabolic activities [279]. The production of various volatile organic compounds (VOCs) by microbial metabolites has garnered significant attention from the global scientific community. These VOCs are the outcomes of the primary and secondary metabolism of soil microorganisms [133]. During primary metabolism, soil microbes generate VOCs as by-products while breaking down food to extract the necessary nutrients required for cell maintenance. In contrast, during secondary metabolism, VOCs are produced by microbes as a response to resource competition in nutrient-poor environments. Recently, several bacterial and fungal strains including *Pseudomonas fluorescens*, *Bacillus mojavensis*, and *Trichoderma* sp. have been reported to produce different types of VOCs [130,132,133]. Microbial VOCs have been extensively documented for their roles as antibacterial, antifungal, and antinematode agents, as well as for promoting plant growth. Additionally, these VOCs function as signaling molecules for cell-to-cell communication between different species. For instance, *Bacillus subtilis* and *Bacillus amyloliquefaciens* produce 2,3-butanediol and acetoin, which play a significant role in promoting plant growth [280]. Other plant-growth-promoting VOCs, like 2-pentylfuran, 13-tetradecadien-1-ol, 2-butanone, and 2-methyl-n-1-tridecene, have been identified in various bacterial strains [281]. VOCs can also trigger induced systemic resistance in plants when faced with pathogen challenges. Notably, volatile compounds such as HCN, dimethyl sulfide, and inorganic volatiles have been found to inhibit growth or have phytotoxic effects [130]. On the other hand, fungi-based VOCs act as location cues for host selection in fungivorous arthropods. For instance, 1-octen-3-ol produced by the wood-rotting white rot fungus Trametes gibbosa attracts fungus-eating beetles (Coleoptera) [131]. Another white rot species, Trametes versicolor, emits sesquiterpenes like d-cadinene, b-guaiene, isoledene, and g-patchoulene, which attract fungivorous beetles in behavioral experiments [131]. Moreover, plants can be protected from biotic stress by treating them with compost supplemented with VOC-producing microbes. The compost acts as a carrier, facilitating the colonization of plant-growth-promoting microbes in the rhizospheric region, subsequently protecting the plant from pathogen attacks by producing VOCs.

## 7. Factors Affecting the Composting Process

The composting process is influenced by several parameters that need to be monitored throughout to ensure the production of high-quality compost. The mechanism of composting is affected by various physico-chemical characteristics of the agricultural feedstocks, including porosity, temperature, oxygen levels, C/N ratio, pH value, proper aeration, and moisture content [15,156,282]. Some of the factors affecting the composting process are described herein.

### 7.1. Temperature and pH Value

Temperature is a key factor which significantly affects the growth and activity of the functional microbial communities in the composting of organic mass (OM). The OM transformation into compost takes place between thermophilic and mesophilic environments. The thermophilic conditions trigger the decomposition of organic waste. The catabolic activities of aerobes help in establishing the ambiance favorable for the decomposition process by shooting up the temperature to 65–70 °C within a few days [283,284]. Hassen et al. (2001) described that the decrease in temperature during composting is associated with the decrease in bacterial population [285]. The pH for the optimal decomposition of the organic waste should also be determined. Conventional composting requires a pH value in the range of 6 to 8 to decompose biomass optimally [286].

### 7.2. Carbon/Nitrogen Ratio

The carbon and nitrogen (C/N) ratio has a major impact on organic waste decomposition during composting. Every organism has a fixed C/N ratio at tissue level. This ratio has a crucial role in deciding the pathway for microbe-mediated decomposition of OM. Carbon serves as an energy source for microorganisms, and hence, it is the basic structural unit of life, whereas nitrogen is an important constituent of proteins, nucleic acids, and other cellular components [287]. Hence, microbes have to be supplemented with carbon-rich materials and nitrogenous sources in a balanced manner. The optimum C/N ratio for rapid composting is 25–35:1. As the carbon content exceeds the optimum amount, the decomposition process becomes progressive [288]. On the other hand, an increase in nitrogen concentration results in unpleasant odor [15]. The regular turning of windrow piles helps in maintaining the C/N ratio by removing the residual nitrogen in ammoniacal form [289].

### 7.3. Quality and Quantity of Incoming Raw Material

The chemical profile of the raw material is very important in the composting process. The low-end product recovery is achieved using organic-content-depleted biomass [156]. Therefore, periodic chemical analysis is required to ensure the quality of the feedstock. The composition of biomass varies as the source varies. Hence, the organic-fraction-rich raw material from fish, vegetable, and fruit markets should be utilized for the composting process [290].

### 7.4. Proper Aeration and Turning

As the biochemical transformation is facilitated by aerobes, proper aeration is needed to ensure oxygen availability. Proper aeration is linked with the periodic turning of the feedstock piles [291]. Turning helps in the smooth running of aerobic compositing processes by evenly distributing the oxygen to every component of the compost pile. Therefore, turning biomass heaps is important in order to maintain adequate aeration [15]. It has been reported that the aerobic composting process is stimulated when a huge amount of oxygen is provided to the project site [292].

### 7.5. Moisture Content

Moisture level is another important factor for the bioconversion process. The optimal range of moisture content is 50 to 55% for organic waste composting [13]. The presence of adequate moisture is crucial for the biochemical activities of microbes facilitating the degradation process. As the moisture exceeds the optimal amount, the environment turns anaerobic as the air spaces are occupied by water, thus reducing oxygen availability to aerobes, whereas a low moisture level will result in the killing of microorganisms [156]. Periodic turning of compost piles not only adjusts the moisture levels but also chops down the large organic matter cakes, and hence, provides microbes with more surface area to attack the material, resulting in a better decomposition process [293].

## 8. Conclusions and Future Directions

In conclusion, as the agricultural industry faces challenges posed by escalating production costs and a growing global population, it is evident that a paradigm shift is needed in the fertilizer sector. The era of the green revolution, once dominated by chemical fertilizers, is gradually fading due to their exorbitant costs and environmental implications. In response to these concerns, the exploration of alternative technologies, such as compost and rhizospheric microorganisms, for sustainable agricultural practices has gained momentum, offering a profitable and eco-friendly solution. Globally, extensive research has been undertaken to investigate the potential of bacterial, cyanobacterial, and fungal biofertilizers for various crops. The application of these biofertilizers has demonstrated positive effects on soil fertility and crop yields in arable lands. Beyond improving nutrient availability, these microorganisms contribute to plant growth through the production of beneficial plant hormones, induction of stress resistance, and biocontrol of plant pathogens. While their performance may vary under different field conditions, the use of microbial fertilizers is anticipated to surge in the foreseeable future due to their economic and ecological benefits.

Looking ahead, the technology of microbial fertilizers can be further enhanced by incorporating sustainable materials as carriers. Utilizing compost as a carrier material, given its sustainability and inherent fertilizing properties, has the potential to elevate biofertilizer technology to the next level. Moreover, in-depth research is required to identify compatible microbial strains capable of thriving in compost and demonstrating optimal physiological performance therein. The application of genome engineering holds potential as a tool for developing superior microbial strains, further advancing the efficacy of biofertilizers. The concept of compost-based biofertilizers represents a novel approach, fostering a unique setting for plant–microbe interactions by harnessing the combined effects of compost and characterized microbial strains. Compost, when combined with a microbial consortium, offers an alternative nutrient source for agricultural plants, while also exhibiting antagonistic effects against soil-borne phytopathogens and inducing tolerance against biotic and abiotic stresses. Furthermore, compost-based biofertilizers not only positively impact soil fertility, but also uphold their safety for use, owing to their eco-friendly nature. Overall, the continuous exploration and development of compost-based biofertilizers, alongside advancements in microbial strain selection and genome engineering, promise to revolutionize modern agriculture, making it more sustainable, economically viable, and environmentally responsible. Embracing these innovations will pave the way for a greener, more productive, and resilient agricultural future.

## Figures and Tables

**Table 2:** Effects of inoculation with PGPR- and AMF-based biofertilizers on the plant, physiological performance under different plant growth promotion mechanisms.

Biofertilizers	Plants	Experiment Sites	Results/Discovery Made	Plant Growth Mechanism	Reference
**PGPR-Based Biofertilizers**					
*Bradyrhizobium* sp.	Wheat (*Triticum aestivum*)	Pot experiment	Results indicate that the inoculation with phosphate biofertilizer significantly enhances grain yield of wheat	IAA production, phosphate solubilization, siderophore production, and HCN production	[162]
*Acinetobacter* sp.	Canola (*Brassica napus*) and Tomato (*Solanum lycopersicum*)	Pot experiment	Result showed that strain inoculation increased the root length and dry biomass of the canola test plants	IAA production, phosphate solubilization, ACC deaminase, and antifungal activity	[163]
*Serratia mercescens*	Wheat (*Triticum aestivum*)	Pot experiment	Result revealed that there was a significant increase in root and shoot lengths, and a significant increase in the root and shoot biomass was also observed	IAA production, siderophore production, and HCN production	[164]
*Pseudomonas striata*	Chickpea (*Cicer arietinum* L.)	Field experiment	It was concluded that there was significant seed grain or crop yield through inoculation with *Pseudomonas striata*	Nitrogen fixation and phosphate solubilization	[165]
*Azotobacter tropicalis*, *Burkhoderia unamae,* and *Bacillus subtilis*	Corn (*Zea mays*)	Pot experiment	Result showed that inoculation significantly enhanced the growth or yield and also increased the fresh and dry weight of corn	Auxin production, nitrogen fixation, phosphate solubilization, and potassium solubilization	[166]
*Azospirillum brasilense* and *Bacillus sphaericus*	Banana (*Musa* spp. cv. ‘*Berangan*’)	Pot and field experiment	Result showed that PGPR inoculation greatly increased the bunch yield	Nitrogen fixation	[167]
*Pseudomonas*, *Rhodococcus,* and *Duganella* sp.	Moss (*Racomitrium japonicum*)	Hydroponic experiment	It was concluded that isolate utilization should promote the moss growth and has potential to be utilized as biofertilizers for moss production	Auxin production, phosphate solubilization, siderophore production, HCN production, and antifungal activity	[168]
*Rhodobacter capsulatus*	Rice (*Oryza sativa* L.)	Field experiment	Results revealed that both biological and grain yields in all the *Rhodobacter capsulatus* inoculated treatments were significantly higher than those in the uninoculated corresponding treatments in both fields	Nitrogen fixation	[168]
*Azotobacter chroococcum* and *Bacillus circulans*	Horseradish Tree (*Moringa oleifera*)	Pot experiment	Result showed that he highest records of shoot and root lengths, and shoot and root dry weights, were obtained with soil inoculation with mixed cultures	IAA production, cytokinin production, nitrogen fixation, phosphate solubilization, and potassium solubilization	[169]
*Azotobacter chroococcum* and *Azospirillum lipoferum*	Coriander (*Coriandrum Sativum*)	Field experiment	Results showed that the highest plant height, umbel number per plant, weight of 1000 seeds, dry weight of plant, and seed yield were obtained by using the biofertilizer twice	Nitrogen fixation and phosphate solubilization	[170]
*Azotobacter* sp., *Nitrobacter* sp., and *Nitrosomonas* sp.	Tomato (*Lycoperscum esculentus*)	Pot experiment	Result indicated that combined biofertilizers are recommended for excellent growth performance of plants	IAA and siderophore production	[171]
*Klebsiella planticola*, *Azotobacter,* and *Bacillus*	Strawberry (*Fragaria × ananassa Duch*)	Pot experiment	A significant effect on yield per plant was noted.	Nitrogen fixation and phytohormones production	[172]
*Azotobacter chroococcum* and *Azospirillum lipoferum*	Ajowan (*Carum copticum*)	Field experiment	It was noted that biological yield, seed yield, essential oil content, and essential oil yield were obtained by using the biofertilizer	Nitrogen fixation	[173]
*Azotobacter chroococcum* and *Bacillus subtilis*	Wheat (*Triticum aestivum* L.)	Field experiment	Organic matrix can be more effective for achieving enhanced productivity of wheat in sub-tropical agro-climatic conditions	Nitrogen fixation, phosphate solubilization, and potassium solubilization	[174]
Rhizobium leguminosarum	Faba bean (*Vicia faba* L.)	Pot experiment	Efficient bioinoculant development to enhance the tolerance of faba bean plants to alkalinity stress and thereby improve the fitness of plants	Nitrogenase activity	[175]
*Acidobacteria* and *Bacteroidetes*	Banana (*Cavendish*)	Pot experiment	Suppressed the strength of *Fusarium* wilt disease through improving soil chemical condition and manipulating the composition of soil microbial community	Nitrogen fixation, phosphate solubilization, and potassium solubilization	[176]
*Bradyrhizobium yuanmingense*	Fabales (*Cajanus cajan* L.)	Field experiment	It was evaluated that their potential as biofertilizers includes being able to replace mineral N fertilization	Nitrogenase activity	[177]
*Acidobacteria*, *Actinobacteria and Proteobacteria*	Banana (*Cavendish*)	Field experiment	Results showed that these bacterial strains are associated with banana Fusarium wilt disease suppression	Antagonistic activity	[178]
*Bacillus licheniformis*	Tomato (*Lycoperscum esculentus*)	Pot experiment	The combined effect of the nitrogen fertilization dose and biofertilizer addition during tomato cultivation on the content of antioxidant compounds in tomato fruits was shown	Nitrogenase activity	[179]
*Rhodopseudomonas palustris*	Rice (*Oryza sativa* L.)	Field experiment	It was concluded that there should be an increase in rice yields in both the organic and saline-flooded paddy fields and concurrently, a reduction in CH4 emissions	Nitrogenase activity	[180]
*Bacillus methylotrophicus*	Tomato (*Lycoperscum esculentus*)	Greenhouse experiment	Results suggest that NKG-1 has potential for commercial application as a biofertilizer or biocontrol agent	Antagonistic activity	[181]
*Streptomyces* *Corchorusii*	Rice (*Oryza sativa* L.)	Pot experiment	Significant enhancement in shoot length and weight of shoot and root, and total grain yield, and weight of grains in rice plants	IAA production, siderophore production, phosphate solubilization, ACC deaminase activity, and antagonistic activity	[182]
*Bacillus thuringiensis*	Tobacco (*Nicotiana*)	Pot experiment	Results revealed that there was reduction in nicotine content and improvement in tobacco quality, which may provide some useful information for tobacco cultivation	Nitrogenase activity	[183]
*Bacillus endophyticus*, *Bacillus sphaericus*, *Enterobacter aerogenes*, *Bacillus safensis,* and *Bacillus megaterium*	Wheat (*Triticum aestivum* L.)	Pot experiment and field experiment	Results revealed that bacterial isolates with plant beneficial traits such as P solubilization are more promising candidates as biofertilizer when used with carrier materials	Phosphate solubilization	[184]
*Bacillus* sp. and *Burkholderia* sp.	Tomato (*Lycoperscum esculentus*)	Pot experiment	The inoculum can tremendously enhance the productivity of tomato, soil fertility, and can also act as a sustainable substitute for chemical fertilizers	IAA production, siderophore production, and phosphate solubilization	[185]
*Azotobacter*	Maize (*Zea mays* L.)	Field experiment	Seed Inoculation has a significant effect on soil properties, growth, and yield of maize	Nitrogen fixation, phosphate solubilization, and potassium solubilization	[186]
*Klebsiella* sp.	Oat seedlings (*Avena sativa*)	Field experiment	Demonstrates that PGPR play an imperative function in stimulating salt tolerance in plants and can be used as biofertilizer to enhance growth of crops in saline areas	Antagonistic activity	[187]
**AMF-Based Biofertilizer**					
*Rhizophagus irregularis*	Pumpkin (*Cucurbita pepo*)	Pot experiment	Results demonstrate that crop inoculation improved the detrimental effect of salinity on plant growth due to improved nutritional contents	Potassium solubilization	[188]
*Glomus versiforme*	Trifoliate orange (*Poncirus trifoliate*)	Pot experiment	AMF inoculation increased the fresh and dry weight and leaf area of seedlings under drought stress	Potassium solubilization and phosphorous solubilization	[189]
*Glomus mosseae*	Cucumber (*Cucumis sativus* L.)	Pot experiment	Results revealed the significance of enhancing plant growth and suppressing damping off of cucumber achieved through inoculation with AMF	Antibiotics production	[190]
*Glomus fasciculatum* and *Gigaspora* sp.	Bean (*Phaseolus*) and Pea (*Pisum sativum*)	Pot experiment	Inoculation led to an increase in shoots and increase in root dry weights	Nitrogen fixation	[191]
*Aspergillus fumigatus* and *Aspergillus niger*	Pigeon pea (*Cajanus cajan* L.)	Pot experiment	Treated plants showed improved nutrient uptake in contrast to non-treated plants	Phosphorous solubilization	[192]
*Funneliformis mosseae*	Tomato (*Solanum lycopersicum*)	Pot experiment	Inoculation confers resistance against salt stress to tomato plants via antioxidant enzymes (POD, CAT, etc.) production	Higher leaf concentration of phosphorous, potassium	[193]
*Fusarium pallidoroseum*	Tomato (*Solanum lycopersicum*), Wheat (*Triticum*), and Maize (*Zea mays*)	Pot experiment	Enhanced proline content, acid and alkaline phosphomonoesterase activity, and peroxidase activity was observed in plants inoculated with *Fusarium pallidoroseum*. The fungus treatment also improved the shoot dry weight and shoot length	Phosphorous solubilization and zinc solubilization	[194]
*Aspergillus niger* and *Penicillium notatum*	Nut (*Arachis hypogaea*)	Pot experiment	Plants showed better nutrient uptake when treated with plant beneficial microbial inoculum	Nitrogen fixation and phosphorous solubilization	[195]
*Acaulospora* spp., *Entrophospora* sp., *Glomus* spp., and *Scutellospora*	Chili (*Capsicum frutescens* L.)	Pot experiment	Highest shoot, root length, and yield were observed in inoculated plants	Nitrogen fixation, phosphorous solubilization, and potassium solubilization	[196]
*Glomus etunicatum* and *Gigaspora* *albida*	Cowpeas (*Vigna unguiculata*)	Pot experiment and field experiment	The treatment resulted in improved grain and shoot biomass production and increased the available P and K levels in soil	Phosphorous solubilization and potassium solubilization	[197]
*Acaulospora laevis*, *Glomus geosporum*, *Glomus mosseae,* and *Scutellospora armeniaca*	Faba bean (*Vicia faba* L.)	Pot experiment	The faba bean inoculated with mixed culture of *Rhizobium* and AMF showed better growth under alkaline environment	Nitrogen fixation and phosphorous solubilization	[175]
*Aspergillus niger*	Chickpea (*Cicer arietinum*)	Pot experiment	The treated plants showed better growth and nutrient profile as compared with non-treated plants	Phosphorous solubilization	[198]
*Glomus fasciculatum*	Pigeon pea (*Cajanus cajan* L.)	Pot experiment	Improved nutrient uptake and biomass production was observed in inoculated pigeon pea plants	Nitrogen fixation, phosphorous solubilization, and potassium solubilization	[199]
*Glomus mosseae*	Garden Pea (*Pisum sativum* L.)	Pot experiment	The plants showed enhanced plant height, leaf area index, and dry matter accumulation when treated with *Glomus mosseae*	Nitrogen fixation and phosphorous solubilization	[200]
*Glomus aggregatum*, *Glomus viscosum*	Tomato (*Solanum lycopersicum* L.)	Pot experiment	The inoculation improved the yield, quality, and nutritional value of tomato plants	Nutrient solubilization	[201]
*Glomus viscosum*	(*Salvia officinalis* L.)	Pot experiment	Better plant growth and biomass production was found in plants treated with fungi	Phosphorous solubilization	[202]
*Glomus intraradices*	Wheat (*Triticum*)	Pot experiment	Enhanced nutrient uptake was observed in inoculated plants	Nutrient solubilization	[203]
*Rhizophagus intraradices*, *Funneliformis mosseae*, and *F. geosporum*	Maize (*Zea mays*)	Pot experiment	Maize treated with AMF showed improved photosynthetic efficacy under high-temperature stress	Nutrient solubilization	[204]

## Data Availability

Not applicable.
